# Development of a Chinese College Students' Attitudes Toward Sexual Swear Words Scale

**DOI:** 10.3389/fpsyg.2021.664065

**Published:** 2021-08-11

**Authors:** Ying Wei, Qingsong Chen

**Affiliations:** ^1^College of Humanities, Zhejiang Normal University, Jinhua, China; ^2^College of International Education, Zhejiang Normal University, Jinhua, China

**Keywords:** sexual swear words, attitude scale, scale development, college student, factor analysis

## Abstract

Sexual swear words are frequently used and considered vulgar and controversial in Chinese. The study of attitude is not only an important part of the study of swear words, but is also an important way for predicting their use. To date, few independent studies have been conducted on Chinese sexual swear words; those that have been conducted mostly focus on language ontology rather than language use. The studies have mainly used qualitative research methods, with a lack of empirical analysis and use of measurement tools. It is feasible and necessary to study college students as the object of sexual swear words because of the prevalence of “Zu'an culture” and the abuse of sexual swear words. Based on the current research status of Chinese swear words and the context of using swear words on campus, this paper combines the research of linguistic differences in swearing, psychological theories, and social science measurement theories and uses SPSS and Mplus statistical software to develop Chinese college students' attitudes toward Sexual Swear Words Scale. The participants consist of students from Zhejiang Normal University and other universities. A total of 262 college students participated in the preliminary test. Through item analysis and exploratory factor analysis, the formal scale was formed. A total of 608 college students were formally tested, and confirmatory factor analysis, reliability, and validity tests were carried out to produce the final scale. The scale contains three subscales: Cognition (17 items), Affection (17 items), and Behavior Tendency (15 items). The results show that each subscale model fits well, has good reliability and validity, and can be used as an important tool to measure attitudes of Chinese college students toward sexual swear words.

## Introduction

Saussure (2017), in *Course in General Linguistics*, pointed out that we should pay attention not only to “correct language” and “beautiful language,” but also to “all forms of expression.” Swear words, as “ inappropriate” and “ungraceful” language, are not only an integral part of daily life, but also an important part of language research. As Li (1994) observed when discussing the importance of studying taboo words, we need to both “understand” and “avoid” certain language forms, neither of which can be separated from investigation and research.

Swear words can be classified into various types according to their sources, constructions, semantic connotations, meanings, swearing subjects, and objects. Studies of Chinese swearing have diverse research topics, encompassing the full range of swear words. For example, Zhou (2006) and Fang (2013) discuss the cognitive problems of animal swear words; Mi (2005) and Chen (2010) analyze death swear words; however, Wang (2005) and Li (2015) discuss clan and couple-addressing swear words. As the “most basic part” (Zhao, 2003), “性丑语” (dirty words about sex) (Yu, 1990) and “性语” (sexual words) (Zhou, 2000) are often used in the category of swear words. However, with the exception of Wang (2005), Jiang and Fan (2008), and Zha and Zhang (2012), few studies have focused on sexual swear words.

Language studies include ontological studies and application studies (Dai, 2015). Studies focusing on Chinese swear word can also be categorized into these two aspects. For example, Liu (2007) and Jiang (2000) focused on the ontological study of Chinese swear words and explored the origin, classification, and function of Chinese swear words in many different periods of time. The other type of research, as exemplified by Zhao (2003), and Zha and Zhang (2012), focuses on swear word use, exploring the cultural and psychological reasons for use by different Chinese groups. However, current research on Chinese swear words mainly focuses on ontology. An insufficient number of research studies have been conducted on the use of swear words; most of the research that has been conducted is based on qualitative methods rather than empirical research. For example, Wang (2006) and Sang (2017) analyzed and empirically summarized the characteristics of swear words in the corpus of A *Dream of Red Mansions* and *Water Margin*. In order to enrich the research field and expand the content and methods of research on Chinese swear words, empirical research needs to be conducted on the use of sexual swear words.

Educational campuses, especially university campuses, are the transmission areas of “Zu'an culture”^1^ (Jiang and Bei, 2020). In a questionnaire survey on sexual swear words, a total of 38 college students were selected randomly by convenience sampling for a questionnaire survey; 27 students (71%) said that they used sexual swear words on a daily basis. Therefore, taking college students as the participants for a study on sexual swear words offers an opportunity to accurately reveal the situation of sexual swear word use and development. Understanding use of sexual swear words by college students will also help to standardize campus language.

Language attitude is closely related to language use, and understanding language attitude helps to gain an understanding of language. In this study, we explored the use and development of sexual swear words from the perspective of attitudes of college student toward them. In macro-level languages and lialects studies, three types of language attitude measurement methods are mainly used: “direct measurement” (Preston, 1999; Anders et al., 2010), “indirect measurement” (Lambert et al., 1960; Agheyisi and Fishman, 1970), and “content analysis of societal treatment” (Garrett, 2010; Soukup, 2012). However, these methods are not fully applicable to micro-level language attitude research such as the study of sexual swear words, making it necessary to construct a new language attitude measurement tool. As a direct measurement method, a questionnaire survey has the advantages of easy operation and conserving human and financial resources, making it suitable for large-scale language attitude surveys (Yu, 2008). This study adopts the questionnaire survey method, using the statistical software SPSS 23.0 and Mplus 8.3 to develop a scale that can be used to measure attitudes toward sexual swear words in universities. The outcome is a data collection tool that can be used for the study of sexual or other types of swear words, providing data support for the study of sexual swear words.

## Definition of the Concept and Dimensions of the Construct

### Definition of Sexual Swear Words and Attitude

#### Definition of Sexual Swear Words

The available definitions of swear words in China and abroad are almost the same and can be divided into three categories. Listed below are several typical definitions:

1. Bad language…means any word or phrase which, when used in what one might call polite conversation, is likely to cause offense (McEnery, 2006, p.1).
2. ST-words are multifunctional, pragmatic units which assume, in addition to the expression of emotional attitudes, various discourse functions (Dewaele, 2004).
3. Swear words are those which: refer to something that is taboo, offensive, impolite, or forbidden in the culture; can be used to express strong emotions, most usually of anger; may evoke strong emotions, most usually of anger or anxiety; include the strongest and most offensive words in a culture—stronger than slang and colloquial language; and may also be used in a humorous way and can be a marker of group identity (Stone et al., 2015).

Definition (1) is based on the semantic features of swear words, including “cause offense,” “inappropriate,” “objectionable,” and “unacceptable.” Stapleton (2010), Beers Fägersten (2012), and Zhang and Qin (2012) all defined swear words in this way. For example, Beers Fägersten (2012) defined swear words as the words “which have the potential to be offensive, inappropriate, objectionable, or unacceptable in any given social context” (p.3).

Definition (2) is based on the semantic functions of swear words. For example, Montagu (1967) and Dewaele (2004) argued that swear words are an expression of emotion. Some Chinese researchers such as Guan (2000) and Meng (2006) used the function of reprimand to define swear words.

Definition (3) uses both semantic features and functions to definite swear words. This type of definitions is the mainstream definition in current research, as supported by Jay (2000, 2009) and Stone et al. (2015), among others.

We take the view that swear words should be defined as per definition (3) and that it is possible to distinguish between broad and narrow swear words. In a broad sense, swear words refer to all words used for swearing, including words, phrases, and sentences. Swear words in a narrow sense refer to the offensive words used to swear, most of which violate social taboos and can easily lead to the swearing objects of the swearing behaviors, or third-party listeners, to feel insulted by the words, phrases, and sentences used. This paper adopts the narrow definition.

Sexual swear words are the swear words related to sex. At present, both the terms and scope of sexual swear words in Chinese and abroad are varied. Currently, Chinese sexual swear word studies mainly use terms such as “性詈语,” (sexual swear words), “性语,” (sexual words), “性丑词” (dirty words about sex), and so on. Some studies even have no terms for sexual swear words, only defining them as part of an abusive or taboo word category. There are also significant differences across studies in the definition of sexual swear words. For example, Jiang (2000) proposed that “sexualized” swear words only relate to male and female sexual organs. Jiang (2007) and Wang (2008) argued that sexual swear words not only relate to sexual organs, but also to include words referring types of sexual behavior and sexual disorder. Jiang and Fan (2008) extended the scope of sexual swear words to incorporate terms from sexual psychology, sexual professions, and other peripheral words related to sex. These categorizations have the following general characteristics: First, most contain swear words related to sexual organs. Although the denotation differs across studies, there is a consensus in most research to classify swear words involving the sexual organs as sexual swear words. Second, there is no unified internal division standard. For example, some studies have classified words relating to sexual organs, sexual behaviors, and abusive lewdness as sexual swear words, which in essence confuse the two criteria of sexual swear word formation and abusive content. Third, the sexual swear word categorization often includes non-sexual words, such as excreta, or words that have no obvious sexual connotation (turtles, etc.). In this study, we redefine the connotation and extension of sexual swear words, building on the previous definitions of sexual swear words. This term “sexual swear words” is used to refer to words, phrases, and sentences that contain sexual organs and behaviors and that can easily lead to abusive feelings among the swearing objects and third-party listeners.

#### Definition of Attitude

The *Contemporary Chinese Dictionary* (7th edition) (Institute of Linguistics, 2016) interprets attitude as a “view of things and actions taken” (p.1266). As an important field in social psychology, study of attitude has a long history. Although there are dozens of different definitions, most social psychologists generally agree that “an attitude is an evaluative reaction to an object” (Dragojevic et al., 2021). However, there is less agreement about the structure of attitudes. Behavioral monism emphasizes the influence of past experience on the formation of attitude and the behavioral significance of attitude, while dualism defines the composition of attitude in terms of cognitive and affective factors. At present, most social psychologists support the “triad theory,” whereby attitude consists of cognition, affection, and behavior tendency (Social Psychology Editorial Team in 13 colleges universities in China, 2003).

After the foundational study of language attitudes by Gardner and Lambert (1972) and Ryan and Giles (1982) language attitude has gradually become an important part of sociolinguistics (Lasagabaster and Gasteiz, 2004). At present, most language attitude definitions are based on the definition of attitude in psychology and have the following characteristics: First, the research objects of the defined language attitude are relatively uniform. Most research usually defines language attitude as attitudes of an individual toward a particular language and its variations, as in definitions (1), (2), and (3) above. Second, there are some differences in the division of dimensions. The dimensionality of linguistic attitude is divided into dualism and trialism. Definition (3) is an example of the dualism division. Hunston and Thompson (2000), Hunston (2003), Feng (2013), and Xie (2015) all divided language attitude into two dimensions, namely, language value and behavior tendency (Xie, 2015). Definition (4) is an example of trialism. White (2000), Martin and White (2005), and Yang and Su (2016) all chose this kind of division. For example, Martin and White (2005) also divided language attitude into appreciation, judgment, and affect.

1. Language attitude is the expression of individuals' social attitude, which focuses specifically on language and its use in society, and by “language” we refer to any kind of language variety (Moreno Fernández, 1998, p.178).
2. Attitudes toward a language (e.g., Hebrew), toward a feature of a language (e.g., a given phonological variant), toward language use (e.g., the use of Hebrew for secular purposes) or toward language as a group marker (e.g., Hebrew as a language of Jews) are all examples of language attitudes (Cooper and Fishman, 1974).
3. Language attitudes refer to evaluative reactions to different language varieties (e.g., accents, dialects) and are organized along two primary evaluative dimensions: status (e.g., intelligent, competent) and solidarity (e.g., friendly, warm) (Dragojevic et al., 2017).
4. Attitude is further divided into appreciation, judgment, and affect (Bednarek, 2009).

Since the existing concept of language attitude mainly focuses on a certain language or variant, it is a macro-level concept. But other micro-level language attitudes should also be included in the study of language attitude. This paper redefines language attitude based on the triad theory of social psychological attitude. Language attitude is defined as a certain structure and relatively stable internal psychological state held by individuals toward a certain language phenomenon or component, which is composed of cognition, affection, and behavior tendency. Therefore, sexual swear words attitude refers to a psychological state with a certain structure, which is relatively stable; it includes cognition, affection, and behavior tendency.

### Preliminary Construction of the Model

To construct a theoretical model of attitudes of Chinese college students toward sexual swear words, it is necessary to confirm its dimensional structure. We established this in two ways: analyzing a corpus of sexual swear words and referring to current linguistic research on sexual swear words and psychological theories.

Following an exhaustive study of the *Contemporary Chinese Dictionary* (Institute of Linguistics, 2016), and searching Chinese literature and online comments, we obtained 27 sexual swear words; we supplemented this with an open-ended questionnaire. We then selected and classified the swear words according to their linguistic characteristics. We call some swear words “non-abusive,” which have tendencies of losing swearing meanings and becoming an interjection or discourse marker. Because the semantic meanings of “sex” have been weakened or are omitted in the process, or at completion, of the words becoming non-abusive, the characters and functions of these words have changed markedly, as is demonstrated by the words “卧槽,” “你 妹,” and “他妈的” (these three words all mean “fuck”). These words do not contain any information about sex, and they are used as interjections or as adverbs of degree most of time, so they need to be excluded from a study of sexual swear words. According to this condition, a total of 19 swear words were obtained. By analyzing their morpheme characteristics, they can be divided into four types of sexual swear words: female sexual organs, male sexual organs, relatives-related sexual behavior, and other sexual behaviors.

Linguistic studies results and psychological theories show that environmental factors are important and affect the use of swear words. Studies by Jay (1992) and Pezdek and Prull (1993) show that there are significant differences in the possibility of using swear words in informal and formal situations, with swear words more likely to be used in relaxed and informal situations. McEnery and Xiao (2004) studied the difference in usage of the word *fuck* and its variants, using the British National Corpus as a data source, and found significant differences in the frequency of swear words such as “Fuck” in scenes with and without interlocutors: it was used more frequently on occasions without interlocutors. Jay and Janschewitz (2008) and Locher and Watts (2005) demonstrated through empirical studies that the context affects assessment of people of the degree to which swear words are offensive, and also affects the possibility of using swear words. Baron and Byrne (2004) study on the cognitive process of impression formation further proved the influence of context on swearing. People often form impressions of others through labor-saving methods such as classifying an individual into a relevant social group. This cognitive process affects the behavior of people in certain situations. Therefore, in unfamiliar and public situations, in order to avoid being directly classified as belonging to a negative image group, swearing behaviors of people are relatively restrained.

Based on the results from the corpus analysis, linguistic research, and psychological theories, we constructed the dimensional structure of attitudes of Chinese college students toward sexual swear words. The Cognition and Affection subscales are divided into four dimensions: female sexual organs, male sexual organs, relatives-related sexual behavior, and other sexual behaviors according to the classification of Chinese sexual swear words. The subscale of Behavior Tendency is constructed according to the difference in swearing situations in linguistic and psychological studies. According to the conditions of whether the occasion is public, whether there are people in the context, and the relationship with others in the context, we divided the subdimensions of behavior tendency into five subdimensions: behavior tendency in public, private, when in a solitary situation, on an Internet platform, and with strangers. The theoretical classification of attitudes of Chinese college students toward sexual swear words is presented in Table 1.

**Table 1:** The dimensional structure of attitude of college students toward sexual swear words.

**Dimension**	**Subdimension**	**Definition**
Cognition	Cognition of female sexual organ swear words	Cognition of the negative attributes of all kinds of sexual swear words
	Cognition of male sexual organ swear words	
	Cognition of relatives-related sexual behavior swear words	
	Cognition of other sexual behavior swear words	
Affection	Affection of female sexual organ swear words	Likes and dislikes of all kinds of sexual swear words
	Affection of male sexual organ swear words	
	Affection of relatives-related sexual behavior swear words	
	Affection of other sexual behavior swear words	
Behavior Tendency	The tendency of swearing in public	The possibility of swearing on various occasions
	The tendency of swearing in private	
	The tendency of swearing in solitary	
	The tendency of swearing on the Internet platform	
	The tendency of swearing in the company of strangers	

## Materials and Methods

### Participants

A total of 993 college students participated in the survey, and 830 valid questionnaires were obtained, which is the sum of sample 1, sample 2, and sample 3. All the participants were L1 Chinese speakers with an L2 English background.

Sample 1: This sample was used to determine the lexical items for the scale through a survey of familiarity of college students with sexual swear words. Students from Zhejiang Normal University were selected to participate in the questionnaire through convenience sampling. A total of 123 questionnaires were distributed, and 112 were valid. The participants consisted of 74 females and 38 males. There were 86 undergraduate students, 11 master's students, and 15 PhD students.

Sample 2: This sample was used for item analysis and exploratory factor analysis. A total of 262 questionnaires were sent out, and 201 valid questionnaires were obtained. The participants consisted of 122 females and 79 males. There were 114 undergraduate students, 72 master's students, and 15 PhD students.

Sample 3: This sample was used for confirmatory factor analysis, validity analysis, and reliability analysis of the formal scale. The sample was asked not to participate in the pretesting. A total of 608 questionnaires were distributed, among which 517 were valid. The participants consisted of 330 females and 197 males. There were 327 undergraduate students, 155 master's students, and 35 Ph.D. students.

### Measures

We produced items for the theoretical model of attitude of Chinese college students toward swear words by means of a presurvey and reference to relevant literature.

First of all, we determined the lexical items needed to produce the items through a presurvey. The most familiar sexual swear words among the four types were obtained *via* a survey of familiarity of college students with sexual swear words. These were then used as representative words of Chinese sexual swear words to produce the items. The previously mentioned 19 sexual swear words were inputted into a questionnaire testing familiarity of college students with sexual swear words. The survey used a Likert scale, with students rating the swear words according to five levels (1 completely unfamiliar with the swear words and 5 very familiar with the swear words). The most familiar sexual swear words were (rating in brackets): “傻逼” (idiot) (4.47), “傻屌” (idiot) (3.93),^2^ “操你妈” (fuck you mother) (4.22), and “日了狗” (fuck) (3.93). Therefore, these four words were used as lexical items for the scale.

Second, we obtained the specific cognitive affection and affectional response of Chinese college students toward sexual swear words through the presurvey and reference to relevant literature, and we determined the specific content of the items. For example, we administered an open-ended questionnaire to ask students about their understanding, motivation, and feelings toward sexual swear words. The answers to the question “What do you think of sexual swear words?” in the open-ended questionnaire were classified into two categories: negative semantic attributes and negative pragmatic functions. The relevant items about negative attributes in the swearing research questionnaires by Janschewitz (2008), Zhang and Li (2016), and Zhang (2018) were referred to for Supplementary Information such as insulting, offensive, and taboo. Finally, the specific content of the cognitive subscale items was determined as “insulting,” “negative,” “impolite,” and another eight items. Similarly, five specific items of the Affection subscale were obtained. The subscale of Behavior Tendency was divided into five items according to the scene types.

A total of 72 items were generated by combining lexical items and specific item types. There were 32 items in the Cognition subscale, 20 items in the Affection subscale, and 20 items in the Behavior Tendency subscale. The items were checked by linguistics teachers and students majoring in psychology, and the item statements had been adjusted according to the review feedback. In order to ensure the quality of sample data, two instructed items were added, based on a 5-point Likert self-rating scale of 1–5, indicating “strongly disagree,” “disagree,” “neutral,” “agree,” and “strongly agree,” respectively. Finally, a 74-item Chinese College Students' Attitude toward Sexual Swear Words Scale was formed.

### Statistical Instruments

A software package SPSS 23.0 was used for item analysis and exploratory factor analysis of the initial scale. Mplus 8.3 was used for confirmatory factor analysis. SPSS 23.0 and Mplus 8.3 were used for validity analysis and reliability analysis of the formal scale.

## Results

### Item Analysis

When comparing the extreme groups of 72 items (except for two instructed items), item 16 (Cognition subscale) was deleted, which did not reach the level of significance (*p* > 0.05). Then, through correlation screening between items and total scores, items 32 (Cognition subscale), 61 and 63 (Behavior Tendency subscale) that had no significant or a low correlation coefficient (<0.40) with the total scores of the subscale were deleted. After item analysis, there were 68 items remaining, including 30 items in the Cognition subscale, 20 items in the Affection subscale, and 18 items in the Behavior Tendency subscale.

### Exploratory Factor Analysis

The results of KMO and Bartlett's test showed that the KMO values of the subscale were 0.92, 0.93, and 0.80, respectively; the results of Bartlett's tests of sphericity were all significant (*p* < 0.001), which was suitable for factor analysis. The items and factors were removed based on the following criteria: (1) an item-to-factor loading lower than 0.45; (2) an item cross-loading >0.40; (3) a commonality value lower than 0.20; (4) an eigenvalue value lower than 1 and containing less than three items; (5) factors that cannot be named or interpreted. An exploratory factor analysis was conducted for each item deleted, and 19 items were finally deleted. The items deleted are as follows:

Items removal from Cognition subscale: 4, 18, 20, 25, 35, 37, 41, 42, 44, 45, 49, 55, 73

Items removal from Affection subscale: 14, 50, 65

Items removal from Behavior Tendency subscale: 36, 48, 68

The final Cognition subscale retained four factors, a total of 17 items, explaining 73.93% variance of the Cognition subscale; the Affection subscale retained three factors, a total of 17 items, explaining 69.38% variance of the Affection subscale; while the Behavior Tendency subscale retained three factors, 15 items in total, explaining 59.69% variance of the Behavior Tendency subscale. Tables 2–4 show the results of exploratory factor analysis.

**Table 2:** Exploratory factor analysis results of Cognition subscale.

**Cognition subscale item**	**Factor loading**			
	**1**	**2**	**3**	**4**
**Factor 1: Cognition of female sexual organ swear words**				
我认为“傻逼”这个词是负面的。 I think the word “傻逼” (idiot) is negative.	**0.82**	0.01	0.28	0.16
我认为“傻逼”这个词是侮辱性的。 I think the word “傻逼” (idiot) is insulting.	**0.81**	0.22	0.24	0.16
我认为“傻逼”这个词是缺乏礼貌的。 I think the word “傻逼” (idiot) is impolite	**0.79**	0.27	0.25	0.14
当我对别人使用“傻逼”一词时,我是在骂他。 When I use the word “傻逼” (idiot) to others, I am scolding them.	**0.77**	0.22	0.07	0.24
当别人对我使用“傻逼”一词时,他是在骂我。 When people use the word “傻逼” (idiot) to me, they are scolding me.	**0.76**	0.13	0.24	0.27
我认为“傻逼”这个词是粗俗的。 I think the word “傻逼” (idiot) is vulgar.	**0.71**	0.36	0.25	0.19
**Factor 2: Cognition of other sexual behavior swear words**				
我认为“日了狗”这个词是缺乏礼貌的 I think the word “日了狗” (fuck) is impolite.	0.17	**0.82**	0.24	0.14
我认为“日了狗”这个词是负面的 I think the word “日了狗” (fuck) is negative.	0.21	**0.82**	0.07	0.14
我认为“日了狗”这个词是侮辱性的 I think the word “日了狗” (fuck) is insulting.	0.22	**0.79**	0.15	0.19
我认为“日了狗”这个词是粗俗的。 I think the word “日了狗” (fuck) is vulgar.	0.16	**0.73**	0.34	0.16
**Factor 3: Cognition of relatives-related sexual behavior swear words**				
我认为“操你妈”这个词是负面的。 I think the word “操你妈” (fuck your mother) is negative.	0.35	0.25	**0.79**	0.12
我认为“操你妈”这个词是缺乏礼貌的。 I think the word “操你妈” (fuck your mother) is impolite.	0.31	0.26	**0.79**	0.06
我认为“操你妈”这个词是侮辱性的。 I think the word “操你妈” (fuck your mother) is insulting.	0.13	0.04	**0.78**	0.27
我认为“操你妈”这个词是粗俗的。 I think the word “操你妈” (fuck your mother) is vulgar.	0.25	0.33	**0.78**	0.13
**Factor 4: Cognition of male sexual organ swear words**				
当我对别人使用“傻屌”一词时,我是在骂他。When I use the word “傻屌”	0.21	0.28	0.15	**0.79**
当别人对我使用“傻屌”一词时,他是在骂我。When people use the word “傻屌” (idiot) to me, they are scolding me.	0.28	0.27	0.24	**0.78**
我使用“傻屌”一词,只是习惯性用法,和普通词汇没有区别。 I use the word “傻屌” (idiot) just in a habitual way. It's no different from ordinary words.	0.20	0.05	0.09	**0.63**

*N = 201. The extraction method was principal components analysis with an orthogonal (varimax) rotations. Factor loadings above 0.45 are in boldface*.

**Table 3:** Exploratory factor analysis results of Affection subscale.

**Affection subscale item**	**Factor loading**		
	**1**	**2**	**3**
**Factor 1: Affection of sexual organ swear words**				
如果有人对我使用“傻逼”一词时,我会生气。 If someone uses the word “傻逼” (idiot) to me, I will be angry.	**0.79**	0.21	0.14
我讨厌别人用“傻逼”一词。 I hate the word “傻逼” (idiot) used by others.	**0.75**	0.25	0.24
对别人使用“傻逼”一词,我有不适感。 I feel uncomfortable when someone use the word “傻逼” (idiot) to others.	**0.75**	0.24	0.20
我对“傻逼”一词很反感。 I'm disgusted with the word “傻逼” (idiot).	**0.75**	0.25	0.17
当听到别人用“傻屌”一词时,我觉得很难受。 It makes me feel bad when people use the word “傻屌” (idiot).	**0.72**	0.29	0.25
如果有人对我使用“傻屌”一词时,我会生气。 If someone uses the word “傻屌” (idiot) to me, I will be angry.	**0.71**	0.18	0.32
对别人使用“傻屌”一词,我有不适感。 I feel uncomfortable when someone use the word “傻屌” (idiot) to others.	**0.67**	0.31	0.34
我对“傻屌”一词很反感。 I'm disgusted with the word “傻屌” (idiot).	**0.61**	0.25	0.31
**Factor 2: Affection of relatives-related sexual behavior swear words**				
我对“操你妈”一词很反感。 I'm disgusted with the word “操你妈” (fuck your mother)	0.30	**0.84**	0.22
当听到别人用“操你妈”一词时,我觉得很难受。 It makes me feel bad when people use the word.“操你妈” (fuck your mother).	0.28	**0.83**	0.15
我讨厌别人使用“操你妈”一词。 I hate the word “操你妈” (fuck your mother) used by others.	0.18	**0.80**	0.145
对别人使用“操你妈”一词,我有不适感。 I feel uncomfortable when someone use the word “操你妈” (fuck your mother) to others.	0.24	**0.76**	0.18
如果有人对我使用“操你妈”一词时,我会生气。 If someone uses the word “操你妈” (fuck your mother) to me, I will be angry.	0.32	**0.73**	0.18
**Factor 3: Affection of other sexual behavior swear words**				
对别人使用“日了狗”一词,我有不适感。 I feel uncomfortable when someone use the word “日了狗” (fuck) to others.	0.23	0.22	**0.84**
我对“日了狗”一词很反感。 I'm disgusted with the word “日了狗” (fuck).	0.34	0.20	**0.82**
我讨厌别人使用“日了狗”一词。 I hate the word “日了狗” (fuck) used by others.	0.20	0.15	**0.81**
如果有人对我使用“日了狗”一词时,我会生气。 If someone uses the word “日了狗” (fuck) to me, I will be angry.	0.33	0.19	**0.69**

*N = 201. The extraction method was principal components analysis with an orthogonal (varimax) rotations. Factor loadings above 0.45 are in boldface*.

**Table 4:** Exploratory factor analysis results of Behavior Tendency subscale.

**Behavior Tendency subscale item**	**Factor loading**		
	**1**	**2**	**3**
**Factor 1: The tendency of swearing in private environments**				
我会在周围只有自己一个人的时候使用“操你妈”一词。 I will use the word “操你妈”” (fuck your mother) in solitary contexts.	**0.79**	0.05	0.04
我会在周围只有自己一个人的时候使用“日了狗”一词。 I will use the word “日了狗” (fuck) in solitary contexts.	**0.75**	−0.07	−0.09
我会在私人场合使用“日了狗”一词。 I will use the word “日了狗” (fuck) in private.	**0.71**	0.18	0.15
我会在周围只有自己一个人的时候使用“傻屌”一词。 I will use the word “傻屌” (idiot) in solitary contexts.	**0.70**	0.15	−0.04
我会在私人场合使用“傻屌”一词。 I will use the word ““傻屌” (idiot) in private.	**0.68**	0.22	0.29
我会在私人场合使用“操你妈”一词。 I will use the word “操你妈” (fuck your mother) in private.	**0.68**	0.09	−0.13
我会在周围只有自己一个人的时候使用“傻逼”一词。 I will use the word “傻逼” (idiot) in solitary contexts.	**0.63**	0.26	0.28
我会在私人场合使用“傻逼”一词。 I will use the word “傻逼” (idiot) in private.^*a*^	**0.60**	0.10	0.26
**Factor 2: The tendency of swearing on the Internet**				
我会在网络公共平台使用“傻屌”一词(微博、Twitter空间等)。 I will use the word “傻屌” (idiot) on the Internet (microblog, Twitter, QQ space, etc.).	0.08	**0.87**	0.15
我会在网络公共平台使用“操你妈”一词(微博、Twitter空间等)。 I will use the word “操你妈 (fuck your mother) on the Internet (microblog, Twitter, QQ space, etc.).	0.02	**0.84**	0.14
我会在网络公共平台使用“傻逼”一词(微博、Twitter空间等)。 I will use the word “傻逼” (idiot) on the Internet (microblog, Twitter, QQ space, etc.).	0.14	**0.69**	0.11
我会在网络公共平台使用“日了狗”一词(微博、Twitter空间等)。I will use the I will use the word “日了狗” (fuck) on the Internet (microblog, Twitter, QQ space, etc.).	0.33	**0.65**	0.13
**Factor 3: The tendency of swearing in public environments**				
我会避免在有陌生人的场合使用“傻逼”一词。 I will avoid using the word “傻逼” (idiot) in the company of strangers.	0.01	0.14	**0.89**
我会避免在公共场合使用“傻屌”一词。 I will avoid using the word “傻屌” (idiot) in public.	0.13	0.16	**0.83**
我会避免在有陌生人的场合使用“操你妈”一词。 I will avoid using the word “操你妈” (fuck your mother) in the company of strangers.	0.05	0.15	**0.71**

a *“傻逼” and “傻屌” both mean idiot, but one is a word about female sexual organ, while another is a word about male sexual organ*.

*N = 201. The extraction method was principal components analysis with an orthogonal (varimax) rotations. Factor loadings above 0.45 are in boldface*.

There were some differences between the results of the exploratory factor analysis and the preliminary theoretical model. The subscale dimensions of Affection and Behavior Tendency changed, and some of the dimensions were merged into the same factor. In the Affection subscale, the Affection of male and female sexual organ swear words were combined into the same factor, which was named as the Affection of sexual organ swear words. In the subscale of Behavior Tendency, private and solitary situations were combined into the same factor, and the situation of public and in the company of strangers were combined into the same factor, which was, respectively, named as tendencies of swearing in a private environment and a public environment.

### Confirmatory Factor Analysis

The scale consists of 49 items and two instructed items. After randomly ranking the items, the Chinese College Students' Attitude toward Sexual Swear Words Scale was formed.

Robust maximum-likelihood estimator in Mplus 8.3 was used to perform a CFA. In addition, we chose χ^2^/df, SRMR, TLI, CFI, and RMSEA as fit indices of the model. The model was modified according to modification indices. The model modification is not only based on the modification indices provided by Mplus, but also in accordance with linguistic theories and practical experience. For example, we established the measurement error in the Behavior tendency subscales e5 and e9 covariant relationship, not only because of modification indices, but also because the measurement indices X5 (I will use the word “傻屌” (idiot) in private) and X9 (I will use the word “傻屌” (idiot) on the Internet) used the same sexual swear word “傻屌” (idiot). There is a practical correlation between the errors, which can establish a covariant relationship. After the model modification, the fit indices of each subscale are presented in Table 5.

**Table 5:** Fit indices of each subscale.

	**χ^2^/df (<3)**	**RMSEA [90% CI] (<0.07)**	**SRMR (<0.08)**	**TLI (>0.92)**	**CFI (>0.92)**
Cognition	2.51	0.054 [0.046 0.062]	0.045	0.95	0.96
Affection	2.64	0.056 [0.049 0.064]	0.037	0.94	0.95
Behavior Tendency	2.43	0.053 [0.043 0.062]	0.048	0.93	0.95

After modification, the fit indices of each subscale were at an acceptable level. There was no negative error variation and no large standard errors in each subscale. The loads of the items were all between 0.50 and 0.95. This CFA model was a good fit.

### Construct Validity and Criterion-Related Validity

#### Construct Validity

The results from the tests on composite reliability (CR), average-variance-extracted (AVE), and the implied correlation coefficients between factors are shown in Tables 6–8.

**Table 6:** CR, AVE, and correlation coefficients between factors in Cognition subscale.

**Dimension**	**CR**	**AVE**	**F1**	**F2**	**F3**	**F4**
F1	0.91	0.63	**0.80**			
F2	0.92	0.73	0.67	**0.86**		
F3	0.89	0.67	0.68	0.64	**0.82**	
F4	0.78	0.54	0.85	0.60	0.54	**0.74**

*The bold figures in the diagonal of the table are the square root of the average-variance-extracted (√AVE)*.

**Table 7:** CR, AVE, and correlation coefficients between factors in Affection subscale.

**Dimension**	**CR**	**AVE**	**F1**	**F2**	**F3**
F1	0.93	0.63	**0.79**		
F2	0.88	0.60	0.70	**0.78**	
F3	0.90	0.69	0.75	0.63	**0.83**

*The bold figures in the diagonal of the table are the square root of the average-variance-extracted (√AVE)*.

**Table 8:** CR, AVE, and correlation coefficients between factors in Behavior Tendency subscale.

**Dimension**	**CR**	**AVE**	**F1**	**F2**	**F3**
F1	0.85	0.41	**0.64**		
F2	0.80	0.50	0.64	**0.70**	
F3	0.71	0.46	0.28	0.48	**0.67**

*The bold figures in the diagonal of the table are the square root of the average-variance-extracted (√AVE), and the non-diagonal figures are the correlation coefficients between the dimensions*.

The CR of the four factors ranged in the Cognition subscale from 0.78 to 0.92, and the AVE of the four factors ranged from 0.54 to 0.73. The CR of the three factors in the Affection subscale ranged from 0.88 to 0.93, and the AVE of the three factors ranged from 0.60 to 0.69. The CR of the three factors in the Behavior Tendency subscale ranged from 0.71 to 0.85, and the AVE of the three factors ranged from 0.41 to 0.50. The AVE of some factors of the Behavior Tendency subscale was smaller than 0.50, but the difference was small. The overall data showed that the Cognition and Affection scales had good convergent validity, while the Behavioral Tendency scale had poor convergent validity, but it was still acceptable.

Comparing the square root value of AVE of each factor with the correlation value of other factors, it was found that the square root value of AVE of each factor in the Affection and Behavior Tendency subscales was greater than that of other factors, and the correlation value of other factors in the Cognition subscale was smaller than that of AVE except for the correlation value between F1 and F4. The results showed that the discriminant validity of the Affection and Behavior Tendency subscales was good, and the discriminant validity of other factors in the Cognition subscales was ideal except for F1 and F4. F1 and F4 were the cognition of swear words about female and male sexual organs, both of which belong to the cognition of sexual organs. Theoretical analysis suggests that there should be a high correlation between them. However, it did not merge into the same factor in the exploratory factor analysis, which may be related to the number of samples.

#### Criterion-Related Validity

The subjects who participated in the formal questionnaire were investigated on their use of sexual swear words. According to their answers, the subjects were divided into two groups: “not using sexual swear words at all” and “using sexual swear words.” A total of 155 students participated in the survey, including 107 females and 47 males. There were 98 undergraduates, 40 master's students and 17 PhD students. Sixty-six participants did not use sexual swear words at all, while 89 did use them. The sexual swear-word user and non-user samples were first tested for normal distribution, and the results showed that the all subscale data of non-users were not normally distributed even after transformation. Therefore, the Mann–Whitney U-test was used for difference analysis in R. The results showed that there were significant differences among the three subscales (*p* < 0.001), which indicated that the three subscales could effectively distinguish between the students who used sexual swear words and those who did not, and had a good criterion-related validity. The 95% confidence intervals, effect sizes, and *p*-valves are shown in Table 9.

**Table 9:** Confidence interval and effect size of each subscale.

	**Difference in ranks [95% CI]**	**Effect size (r) [95% CI]**	***p*-value**
Cognition	−10.00 [−14.00 −7.00]	−0.43 [−0.54 −0.29]	1.181e-08
Affection	−13.00 [−16.00 −9.00]	−0.49 [−0.60 −0.38]	5.997e-11
Behavior Tendency	−11.00 [−14.00 −8.00]	−0.46 [−0.58 −0.33]	6.755e-10

### Internal Consistency Reliability

The Cronbach's α coefficients of the Cognition, Affection, and Behavior Tendency subscales were 0.93, 0.95, and 0.87, respectively. The Cronbach's α coefficients of the four Cognition subscale factors were 0.91, 0.92, 0.89, and 0.76, respectively. The Cronbach's α coefficients of the three Affection subscale factors were 0.93, 0.89, and 0.90, respectively. The Cronbach's α coefficients of the three factors in the Behavior Tendency subscale were 0.85, 0.79, and 0.70, respectively. The Cronbach's α coefficients were all >0.70, and almost all other coefficients were >0.80 except for the Behavior Tendency subscale, which indicates that these subscales have good reliability and high internal consistency.

## Discussion

Based on the dimensional structure of attitudes of Chinese college students toward sexual swear words, this study developed a Chinese College Students' Attitudes toward Sexual Swear Words Scale. After the item analysis, exploratory factor analysis, confirmatory factor analysis, reliability and validity tests, a formal Chinese College Students' Attitudes toward Sexual Swear Words Scale was obtained. The formal scale has a total of 49 items, which is composed of three subscales, namely, Cognition, Affection, and Behavior Tendency. There are 17 items in the Cognition subscale, which could explain 73.93% variance of the Cognition subscale; 17 items in the Affection subscale, which could explain 69.38% variance of the Affection subscale; and 15 items in the Behavior Tendency subscale, which could explain 59.69% variance of the Behavior Tendency subscale. Confirmatory factor analysis showed that the factors of each subscale fit well. The reliability analysis showed that the Cronbach's α coefficient of the subscales and the internal factors of each subscale ranged from 0.70 to 0.95, and the reliability was good. The CR of subscales and the combination of factors within each subscale ranged from 0.71 to 0.92, and the AVE ranged from 0.50 to 0.73, except for the Behavior Tendency subscale, which were slightly lower than 0.50. The square root of the AVE was higher than the correlation value among other factors except for two factors in the Cognition subscale, which showed good construct validity. At the same time, there were significant differences in the scores of each subscale between the students who used sexual swear words and those who did not use them. Therefore, this scale is an effective tool to measure attitudes of Chinese college students toward sexual swear words.

The development of the Chinese College Students' Attitudes toward Sexual Swear Words Scale is a simple attempt based on current research into Chinese swear words and the current status of use of swear words by Chinese college students. This paper takes sexual swear words as the research object, attitude as the research content, and expands the research on sexual swear words and their use. Empirical research methods, using measurement and statistics, were adopted for the study of sexual and other swear words. The Chinese College Students' Attitudes toward Sexual Swear Words Scale can also provide data support for language planning.

However, there are still several limitations in the process of scale development. First, the sampling method can be further improved. In this study, due to limitations in the survey conditions, convenience sampling was adopted to obtain participant samples. In the future, stratified random sampling and other methods could be used to further improve sample representativeness. Second, the sample size for exploratory factor analysis is small. Although the sample scale used for exploratory factor analysis was more than five times greater than the number of items and the total number reached 200, which meets the standard sample size for exploratory factor analysis, the factor analysis results would be more stable if the sample size were to be increased. Third, there are too many negatively worded items. Wu (2010) argues that too many negatively worded items are likely to affect the results of factor analysis, which brings a lot of inconvenience to the construct analysis. The low AVE of the Behavior Tendency subscale and poor convergent validity may also be attributed to this. Fourth, the research object needs to be further broadened. In this paper, sexual swear words are defined as those related to sex that have not reached a state of full completion as non-abusive terms. Therefore, many obvious non-abusive sexual swear words were not included in the study. However, these words are an important part of the development and change of sexual swear words and have high linguistic value. It is hoped that these limitations can be continuously addressed in the follow-up studies and that the scale will provide a scientific research tool for the empirical study of sociolinguistic swear words.

## Data Availability Statement

The original contributions presented in the study are included in the article/Supplementary Material, further inquiries can be directed to the corresponding author.

## Ethics Statement

Ethical review and approval was not required for the study on human participants in accordance with the local legislation and institutional requirements. Written informed consent for participation was not required for this study in accordance with the national legislation and the institutional requirements.

## Author Contributions

YW wrote the initial draft of the manuscript. QC advised on the structure and revised the manuscript. All authors contributed to the article and approved the submitted version.

## Conflict of Interest

The authors declare that the research was conducted in the absence of any commercial or financial relationships that could be construed as a potential conflict of interest.

## Publisher's Note

All claims expressed in this article are solely those of the authors and do not necessarily represent those of their affiliated organizations, or those of the publisher, the editors and the reviewers. Any product that may be evaluated in this article, or claim that may be made by its manufacturer, is not guaranteed or endorsed by the publisher.

## Footnotes

^1^“Zu'an” originally referred to the “zu'an” region of a competitive online game server in China. It is rumored that game players in this region like to swear the most, and so “zu'an” gradually become a byword for swearing.

^2^“傻逼” and “傻屌” both mean idiot, but one is a word about female sexual organ, while another is a word about male sexual organ.

## References

[B1] Agheyisi R. Fishman J. A. (1970). Language attitude studies: a brief survey of methodological approaches. Anthropol. Linguist. 12, 137–157.

[B2] Anders C. A. Hundt M. Lasch A. (2010). Perceptual Dialectology. Amsterdam: de Gruyter.

[B3] Baron R. A. Byrne D. (2004). Social Psychology (Huang, M. Trans). Shanghai: East China Normal University Press.

[B4] Bednarek M. (2009). Language patterns and ATTITUDE. Funct. Lang. 16, 165–192. 10.1075/fol.16.2.01bed

[B5] Beers Fägersten K. A. (2012). Who's Swearing Now: The Social Aspects of Conversational Swearing. Newcastle: Cambridge Scholars Publishing.

[B6] Chen W. (2010). On the swear words of “death” in Shantou dialect (Qiantan shantouhua “siwanglei” liyu). Youth Writ. 17, 175–176.

[B7] Cooper R. L. Fishman J. A. (1974). The study of language attitudes. Int. J. Sociol. Lang. 3, 5–20.

[B8] Dai Q. (2015). The status of language use research in the field of sociolinguistics. J. Chin. Linguist. 2, 1–8.

[B9] Dewaele J. (2004). The emotional force of swearwords and taboo words in the speech of multilinguals. J. Multiling. Multicult. Dev. 25, 204–222. 10.1080/01434630408666529

[B10] Dragojevic M. Fasoli F. Rakić T. (2021). Toward a century of language attitudes research: looking back and moving forward. J. Lang. Soc. Psychol. 40, 60–79. 10.1177/0261927X20966714

[B11] Dragojevic M. Giles H. Beck A. C. Tatum N. T. (2017). The fluency principle: why foreign accent strength negatively biases language attitudes. Commun. Monogr. 84, 385–405. 10.1080/03637751.2017.1322213

[B12] Fang Z. (2013). A cognitive linguistic study of Chinese animal swear words (Hanyu dongwulei lima ciyu de renzhi yuyanxue yanjiu) (master's thesis). Yunnan Normal University, Kunming, China.

[B13] Feng G. (2013). On three performances of the language attitude. Linguist. Study 33, 112–118.

[B14] Gardner R. C. Lambert W. E. (1972). Attitudes and Motivation in Second Language Learning. Rowley, MA: Newbury House.

[B15] Garrett P. (2010). Attitudes to Language. New York, NY: Cambridge University Press.

[B16] Guan E. (2000). The cultural meanings of animal terms in Swearwords (Liyu zhong dongwu ciyu de wenhua hanyi). J. Guangxi Normal Univ. 1, 40–43. 10.16088/j.issn.1001-6597.2000.01.009

[B17] Hunston S. (2003). “Frame, phrase or function: a comparison of frame semantics and local grammars,” in Corpus Linguistics 2003 (Technical Papers 16), eds D. Archer, P. Rayson, A. Wilson, and T. McEnery (Lancaster: University Centre for Computer Corpus Research on Language), 342–358.

[B18] Hunston S. Thompson G. (2000). Evaluation in Text: Authorial Stance and the Construction of Discourse. Oxford: Oxford University Press.

[B19] Institute of Linguistics CASS. (2016). The Contemporary Chinese Dictionary, 7th Edn. Beijing: The Commercial Press.

[B20] Janschewitz K. (2008). Taboo, emotionally valenced, and emotionally neutral word norms. Behav. Res. Methods 40, 1065–1074. 10.3758/BRM.40.4.1065 19001397

[B21] Jay T. (1992). Cursing in America: A Psycholinguistic Study of Dirty Language in the Courts, in the Movies, in the Schoolyards and on the Streets. Philadelphia, PA: John Benjamins Publishing Company.

[B22] Jay T. (2000). Why We Curse: A Neuro-Psycho-Social Theory of Speech. Amsterdam: John Benjamins Public Company.

[B23] Jay T. (2009). The utility and ubiquity of taboo words. Perspect. Psychol. Sci. 4, 153–161. 10.1111/j.1745-6924.2009.01115.x 26158942

[B24] Jay T. Janschewitz K. (2008). The pragmatics of swearing. J. Polit. Res. Lang. Behav. Cult. 4, 267–288. 10.1515/JPLR.2008.013

[B25] Jiang F. Bei H. (2020). Vulgar and Violent, Swear Words Has Become a Subculture, “Zuan Culture” Is Eroding the Campus. Beijing: China Comment. Available online at: https://mp.weixin.qq.com/s/WJgsgiA45P_7xX9oSgioGw (accessed July 13, 2020).

[B26] Jiang J. (2000). The formation and analysis of abusive language. J. Anq. Teach. Coll. 1, 101–104.

[B27] Jiang M. (2007). Study on Chinese swear words (master's thesis). Jilin University, Changchun, China.

[B28] Jiang Z. Fan R. (2008). A psychological and cultural analysis of Chinese sexual swear words (Hanyu xingliyu de xinli ji wenhua fenxi). Qinghai Soc. Sci. 3, 192–195. 10.3969/j.issn.1001-2338.2008.03.048

[B29] Lambert W. E. Hodgson R. C. Gardner R. C. Fillenbaum S. (1960). Evaluational reactions to spoken languages. J. Abnorm. Soc. Psychol. 60, 44–51. 10.1037/h0044430 14413611

[B30] Lasagabaster D. Gasteiz V. (2004). “Attitude/Einstellung,” in Sociolinguistics An International Handbook of the Science of Language and Society, 2nd ed., Vol. 1., eds U. Ammon, N. Dittmar, K. J. Mattheier, and P. Trudgill (Berlin: Walter de Gruyter), 399–405.

[B31] Li R. (1994). Examples of taboo words (Jinjizi juli). Dialect 3, 161–169.

[B32] Li X. (2015). The semantic evolution and its causes of swear words of wife appellation in Shenqiu dialect (Shenqiu fangyan qizu chengwei limayu de yuyi yanbian ji qi chengyin). Seeker 7, 174–178.

[B33] Liu F. (2007). A brief history of ancient Chinese abusive language (Ph.D. thesis). Zhejiang University, Hangzhou, China.

[B34] Locher M. A. Watts R. J. (2005). Politeness theory and relational work. J. Polit. Res. Lang. Behav. Cult. 1, 9–33. 10.1515/jplr.2005.1.1.9

[B35] Martin J. R. White P. R. R. (2005). The Language of Evaluation Appraisal in English. Houndmill: Palgrave Macmillan.

[B36] McEnery A. Xiao Z. (2004). Swearing in modern British English: the case of fuck in the BNC. Lang. Lit. 13, 235–268. 10.1177/0963947004044873

[B37] McEnery T. (2006). Swearing in English Bad Language, Purity, and Power From 1586 to the Present. New York, NY: Routledge.

[B38] Meng Z. (2006). The mode of being dirty words and the cultural connotation about Chinese dirty words. Qilu J. 4, 77–81.

[B39] Mi M. (2005). Study on abusive language. J. Binzhou Univ. 21, 55–57.

[B40] Montagu A. (1967). The Anatomy of Swearing, 1973 Edn. London: MacMillan.

[B41] Moreno Fernández F. (1998). Principios de sociolingüí*stica y sociología del lenguaje [Principles of sociolinguistics and language sociology]*. Barcelona: Ariel.

[B42] Pezdek K. Prull M. (1993). Fallacies in memory for conversations: reflections on Clarence Thomas, Anita Hill, and the Like. Appl. Cogn. Psychol. 7, 299–310. 10.1002/acp.2350070404

[B43] Preston D. R. (ed.). (1999). “A language attitude approach to the perception of regional variety,” in Handbook of perceptual dialectology (Amsterdam; Philadelphia, PA: John Benjamins).

[B44] Ryan E. B. Giles H. (1982). Attitudes Towards Language Variation. London: Arnold.

[B45] Sang Z. (2017). The types, characteristics and acceptance of swearing in the romance of the Three Kingdoms (Sanguoyanyi maliyu de leixing tese he jieshou). J. Ming Qing Fict. Stud. 4, 82–96. 10.13674/j.cnki.32-1017/i.2017.04.006

[B46] Saussure F. D. (2017). Course in General Linguistics (Gao, M., Trans). Beijing: The Commercial Press.

[B47] Social Psychology Editorial Team in 13 colleges and universities in China (2003). Social Psychology. Tianjin: Nankai University Press.

[B48] Soukup B. (2012). Current issues in the social psychological study of‘language attitudes’: constructionism, context,and the attitude–behavior link. Lang. Linguist. Compass 4, 212–224. 10.1002/lnc3.332

[B49] Stapleton K. (2010). “Swearing,” in Interpersonal Pragmatics, eds M. A. Locher, and S. L. Graham (Mouton: De Gruyter), 289–305.

[B50] Stone T. E. McMillan M. Hazelton M. (2015). Back to swear one: a review of English language literature on swearing and cursing in Western health settings. Aggress. Violent Behav. 25, 65–74. 10.1016/j.avb.2015.07.012

[B51] Wang Q. (2006). A study of swearing in a dream of Red Mansions (master's thesis). Southwest University, Chongqing, China.

[B52] Wang X. (2008). Classification and cultural meaning of swearing words in the dream of the red chamber. J. Ningbo Inst. Educ. 4, 50–54. 10.3969/j.issn.1009-2560.2008.04.013

[B53] Wang Y. (2005). Cultural interpretation of clan and swear words (Zongzu ji xingliyu de wenhua chanshi). J. Ningxia Univ. 27, 37–39. 10.3969/j.issn.1001-5744.2005.02.007

[B54] White P. R. R. (2000). “Appraisal - the language of evaluation and stance,” in Handbook of Pragmatics, eds J. Verschueren, J. Östman, J. Blommaert, and C. Bulcaen (Amsterdam: John Benjamins), 1–27.

[B55] Wu M. (2010). Structural Equation Modeling: Tips for Operation and Application (Jiegou Fangcheng Moxing: Amos de caozuo yu yingyong). Chongqing: Chongqing University Press.

[B56] Xie Y. (2015). A survey on language attitude towards the public service in Macau. Appl. Linguist. 2, 19–28. 10.16499/j.cnki.1003-5397.2015.02.003

[B57] Yang W. Su J. (2016). An investigation of the language attitudes towards Chinese of international students learning Chinese as a second language. J. Tianjin Norm. Univ. 5, 67–71.

[B58] Yu Q. (2008). A comparison of two research techniques in language attitude studies: evidence from an investigation on senior high school students in Changzhou. J. Chin. Sociolinguist. 1, 128–137.

[B59] Yu Y. (1990). Swear Words (Chouyu Daguan). Shijiazhuang: Hebei People Publishing House.

[B60] Zha X. Zhang J. (2012). Social and cultural psychological analysis of “Ta ma de” (Guoma de shehui wenhua xinli fenxi). Guizhou Soc. Sci. 2, 19–23.

[B61] Zhang Q. (2018). A study about college students' usage degree and harmfulness of Chinese abusive language. J. Guiyang Univ. 13, 53–57. 10.3969/j.issn.1673-6133.2018.03.010

[B62] Zhang Q. Li X. (2016). A study on the concept structure of Chinese swear words (Hanyu liyu de gainian jiegou yanjiu). J. South China Norm. Univ. 3, 93–102.

[B63] Zhang Y. Qin Z. (2012). On the origin of swearing culture in films and TV dramas in Northeast China (Dongbei yingshi chuanmei lima yuyan wenhua tanyuan). Soc. Sci. Front Bimonthly 10, 263–264.

[B64] Zhao R. (2003). Language and Gender (Yuyan Yu Xingbie). Shanghai: Shanghai Foreign Language Education Press.

[B65] Zhou R. (2000). A Study of Chinese Swear words (master's thesis). Beijing: Beijing Language and Culture University.

[B66] Zhou Y. (2006). On the cultural and psychological reasons for the derogatory and appreciative sense of Chinese phrases with animal words. Lang. Teach. Linguist. Stud. 2, 43–47.

