# Supplementary material for: Development of a Chinese College Students' Attitudes Toward Sexual Swear Words Scale
Source: Front Psychol. 2021 Aug 11;12:664065. doi: 10.3389/fpsyg.2021.664065 (PMC8385270; doi:10.3389/fpsyg.2021.664065)
Supplement: Supplementary file 1 [file Data_Sheet_1.pdf]

Appendix

大学生性詈语态度认知分量表　Cognition Subscale

我认为“傻逼”这个词是负面的。 I think the word “傻逼” (idiot) is negative.

我认为“傻逼”这个词是侮辱性的。 I think the word “傻逼” (idiot) is insulting.

我认为“傻逼”这个词是缺乏礼貌的。 I think the word “傻逼” (idiot) is impolite

当我对别人使用“傻逼”一词时，我是在骂他。 When I use the word “傻逼” (idiot) to others, I am scolding them.

当别人对我使用“傻逼”一词时，他是在骂我。 When people use the word “傻逼” (idiot) to me, they are scolding me.

我认为“傻逼”这个词是粗俗的。 I think the word “傻逼” (idiot) is vulgar.

我认为“日了狗”这个词是缺乏礼貌的。 I think the word “日了狗” (fuck) is impolite.

我认为“日了狗”这个词是负面的。 I think the word “日了狗” (fuck) is negative.

我认为“日了狗”这个词是侮辱性的。 I think the word “日了狗” (fuck) is insulting.

我认为“日了狗”这个词是粗俗的。 I think the word “日了狗” (fuck) is vulgar.

我认为“操你妈”这个词是负面的。 I think the word “操你妈” (fuck your mother) is negative.

我认为“操你妈”这个词是缺乏礼貌的。 I think the word“操你妈” (fuck your mother) is impolite.

我认为“操你妈”这个词是侮辱性的。 I think the word “操你妈” (fuck your mother) is insulting.

我认为“操你妈”这个词是粗俗的。 I think the word “操你妈” (fuck your mother) is vulgar.

当我对别人使用“傻屌”一词时，我是在骂他。 When I use the word “傻屌” (idiot) to others, I am scolding them.

当别人对我使用“傻屌”一词时，他是在骂我。 When people use the word “傻屌” (idiot) to me, they are scolding me.

我使用“傻屌”一词，只是习惯性用法，和普通词汇没有区别。 I use the word “傻屌” (idiot) just in a habitual way. It’s no different from ordinary words.

大学生性詈语态度情感分量表 Affection Subscale

如果有人对我使用“傻逼”一词时，我会生气。 If someone uses the word “傻逼” (idiot) to me, I will be angry.

我讨厌别人用“傻逼”一词。 I hate the word “傻逼” (idiot) used by others.

对别人使用“傻逼”一词，我有不适感。I feel uncomfortable when someone use the word “傻逼” (idiot) to others.

我对“傻逼”一词很反感 I’m disgusted with the word “傻逼” (idiot).

当听到别人用“傻屌”一词时，我觉得很难受。 It makes me feel bad when people use the word “傻屌” (idiot).

如果有人对我使用“傻屌”一词时，我会生气。 If someone uses the word “傻屌” (idiot) to me, I will be angry.

对别人使用“傻屌”一词，我有不适感。I feel uncomfortable when someone use the word “傻屌” (idiot) to others.

我对“傻屌”一词很反感。 I'm disgusted with the word “傻屌” (idiot).

我对“操你妈”一词很反感。 I’m disgusted with the word “操你妈” (fuck your mother).

当听到别人用“操你妈”一词时，我觉得很难受。 It makes me feel bad when people use the word “操你妈” (fuck your mother).

我讨厌别人使用“操你妈”一词。 I hate the word “操你妈” (fuck your mother) used by others.

对别人使用“操你妈”一词，我有不适感。 I feel uncomfortable when someone use the word “操你妈” (fuck your mother) to others.

如果有人对我使用“操你妈”一词时，我会生气。If someone uses the word “操你妈” (fuck your mother) to me, I will be angry.

对别人使用“日了狗”一词，我有不适感。 I feel uncomfortable when someone use the word “日了狗” (fuck) to others.

我对“日了狗”一词很反感。 I’m disgusted with the word “日了狗” (fuck).

我讨厌别人使用“日了狗”一词。 I hate the word “日了狗” (fuck) used by others.

如果有人对我使用“日了狗”一词时，我会生气。If someone uses the word “日了狗” (fuck) to me, I will be angry.

大学生性詈语态度行为倾向分量表 Behavior Tendency Subscale

我会在周围只有自己一个人的时候使用“操你妈”一词。I will use the word “操你妈” (fuck your mother) in solitary contexts.

我会在周围只有自己一个人的时候使用“日了狗”一词。 I will use the word “日了狗” (fuck) in solitary contexts.

我会在私人场合使用“日了狗”一词。 I will use the word “日了狗”(fuck) in private.

我会在周围只有自己一个人的时候使用“傻屌”一词。 I will use the word “傻屌” (idiot) in solitary contexts.

我会在私人场合使用“傻屌”一词。 I will use the word “傻屌” in private.

我会在周围只有自己一个人的时候使用“傻逼”一词。 I will use the word “傻逼” (idiot) in solitary contexts.

我会在私人场合使用“操你妈”一词。 I will use the word “操你妈” (fuck your mother) in private.

我会在私人场合使用“傻逼”一词。 I will use the word “傻逼” (idiot) in private.

我会在网络公共平台使用“傻屌”一词（微博、Twitter、空间等）。 I will use the word “傻屌” (idiot) on the Internet (microblog, Twitter, QQ space, etc.).

我会在网络公共平台使用“操你妈”一词（微博、Twitter、空间等）。 I will use the word “操你妈” (fuck your mother) on the Internet (microblog, Twitter, QQ space, etc.).

我会在网络公共平台使用“傻逼”一词（微博、Twitter、空间等）。 I will use the word “傻逼” (idiot) on the Internet (microblog, Twitter, QQ space, etc.).

我会在网络公共平台使用“日了狗”一词（微博、Twitter、空间等）。I will use the word “日了狗” (fuck) on the Internet (microblog, Twitter, QQ space, etc.).

我会避免在有陌生人的场合使用“傻逼”一词。I will avoid using the word “傻逼” (idiot) in the company of strangers.

我会避免在公共场合使用“傻屌”一词。 I will avoid using the word “傻屌” (idiot) in public.

我会避免在有陌生人的场合使用“操你妈”一词。 I will avoid using the word “操你妈” (fuck your mother) in the company of strangers.
